# DYRK1B blocks canonical and promotes non-canonical Hedgehog signaling through activation of the mTOR/AKT pathway

**DOI:** 10.18632/oncotarget.13662

**Published:** 2016-11-26

**Authors:** Rajeev Singh, Pavan Kumar Dhanyamraju, Matthias Lauth

**Affiliations:** ^1^ Philipps University Marburg, Institute of Molecular Biology and Tumor Research (IMT), Center for Tumor- and Immunobiology, 35043 Marburg, Germany

**Keywords:** hedgehog, GLI1, DYRK1B, MIRK, AKT

## Abstract

Hedgehog (Hh) signaling plays important roles in embryonic development and in tumor formation. Apart from the well-established stimulation of the GLI family of transcription factors, Hh ligands promote the phosphorylation and activation of mTOR and AKT kinases, yet the molecular mechanism underlying these processes are unknown. Here, we identify the DYRK1B kinase as a mediator between Hh signaling and mTOR/AKT activation. In fibroblasts, Hh signaling induces DYRK1B protein expression, resulting in activation of the mTOR/AKT kinase signaling arm. Furthermore, DYRK1B exerts positive and negative feedback regulation on the Hh pathway itself: It negatively interferes with SMO-elicited canonical Hh signaling, while at the same time it provides positive feed-forward functions by promoting AKT-mediated GLI stability. Due to the fact that the mTOR/AKT pathway is itself subject to strong negative feedback regulation, pharmacological inhibition of DYRK1B results in initial upregulation followed by downregulation of AKT phosphorylation and GLI stabilization. Addressing this issue therapeutically, we show that a pharmacological approach combining a DYRK1B antagonist with an mTOR/AKT inhibitor results in strong GLI1 targeting and in pronounced cytotoxicity in human pancreatic and ovarian cancer cells.

## INTRODUCTION

Hedgehog (Hh) signaling is an important regulatory system in embryonic development, stem cell biology and tumorigenesis [1–3]. Mechanistically, Hh ligands (Sonic Hh (SHH), Indian Hh (IHH), Desert Hh (DHH)) bind to Patched (PTCH1, PTCH2) receptors, thereby de-repressing the transmembrane protein Smoothened (SMO). Activated SMO results in the generation of transcriptionally competent forms of the transcription factors GLI2 and GLI3, which enter the nucleus and initiate target gene expression. Well-established target genes include e.g. *PTCH1* and *GLI1*, which regulate the Hh pathway in a negative and positive manner, respectively, and are often utilized as surrogate read-outs for general pathway activity. Such transcriptional feed-back loops are frequently encountered in physiologically important signaling pathways and serve to fine-tune the entire system. In addition, non-transcriptional regulatory inputs through e.g. kinases have been documented and it is well known that Hh signaling promotes the phosphorylation of e.g. MEK, ERK and AKT kinases [4–7]. These mechanisms are often exploited in cancer cells in ‘non-canonical’ modes of signaling, leading to e.g. Hh ligand/receptor-independent activation of GLI transcription factors [8–11].

One group of enzymes with close regulatory connection to the Hh pathway is the DYRK (Dual-specificity and Tyrosine(Y)-regulated kinase) kinase family with its five members DYRK1A, DYRK1B, DYRK2, DYRK3 and DYRK4 [12–17]. In particular, DYRK1B (a.k.a. MIRK) is linked to the development of several cancer types and can frequently be found amplified or hyperactive in ovarian and in pancreatic cancer [18–23]. While some studies described a negative role for DYRK1B in Hh signal transduction [14, 24, 25], others have documented a stimulatory function for this kinase [15]. In our present study, we aimed to shed light on this issue and to clarify the role of DYRK1B in Hh signaling. We could observe that DYRK1B has opposing roles on the Hh output depending on the site of integration in the pathway: While it suppresses canonical (membrane-initiated) Hh signaling, it promotes the non-canonical (downstream) stabilization of GLI1. The latter effect is mediated by the hitherto unknown ability of DYRK1B to activate the PI3K/mTOR/AKT pathway, which is known to stabilize GLI proteins [7, 26]. The PI3K/mTOR/AKT pathway is one of the most frequently activated signaling cascades in human cancer [27]. The mTOR kinase can be found in at least two multi-protein complexes, termed mTORC1 and mTORC2. The first complex is downstream of AKT and is activated through TSC1/2 and RHEB proteins whereas the latter complex is upstream of AKT and is activated by PI3K in an unknown manner [28]. The mTORC1 complex is well established for being a major regulator of protein translation and autophagy while mTORC2, amongst others, impinges on cell survival through regulation of AKT, FOXO and PKCα [29, 30].

Here, we present data that endogenous Hh signaling augments DYRK1B levels, and that blocking this increase abrogates the Hh-induced stimulation of mTOR/AKT signaling. The intense crosstalk between Hh signaling, GLI and mTOR/AKT is further complicated by mTOR/AKT being subject to strong feedback control through mTORC1 and S6K. In turn, we observed that blocking DYRK1B function by RNAi or small molecule inhibition resulted in a time-dependent impact on GLI1 levels and Hh pathway output. Continuing from these mechanistic findings, we could furthermore demonstrate that a pharmacological therapy combining the targeted inhibition of DYRK1B with that of PI3K/mTOR/AKT has strong effects on Hh/GLI signaling and on cell growth of *DYRK1B*-amplified pancreatic and ovarian cancer cells.

## RESULTS

### Differential effects of DYRK1B on Hh signaling

**Figure 1:**
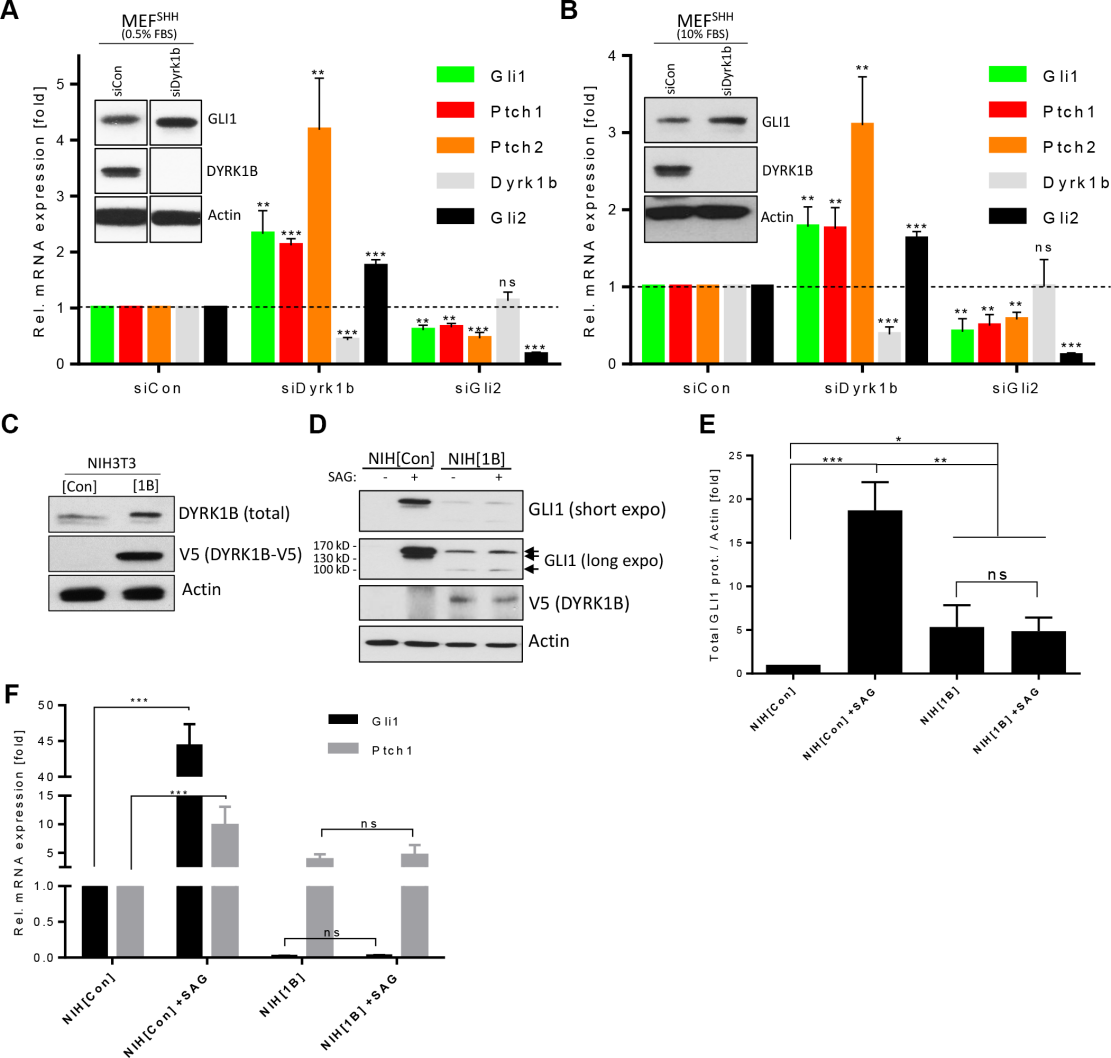
Differential effects of DYRK1B on Hh/GLI signaling (**A**) Hh target gene expression in siRNA-transfected mouse embryonic fibroblasts stably expressing SHH (MEF^[SHH]^). Shown is the mean ± SD of *n* = 3. Cells were cultured in 0.5% FBS-containing media. The inset shows a Western blot of the same experiment (samples were run on the same membrane with intervening lanes cut out). ns = non significant. (**B**) The same experiment as in (A), but performed in 10% FBS-containing media. (**C**) Immunoblot of lysates from NIH3T3 cells stably harboring an empty control (mock; NIH^[Con]^) or a *DYRK1B-V5* (NIH^[1B]^) expression plasmid. (**D**) Western analysis of NIH^[Con]^ and NIH^[1B]^ cells treated with SAG (100 nM) for 48 h. Note the three different GLI1 isoforms (arrows). (**E**) Quantification of the sum of all GLI1 bands (normalized against Actin) depicted in (D). Shown is the mean ± SD of *n* = 3. (**F**) Quantitative PCR of Hh target gene expression (*Gli1, Ptch1*) in SAG-treated NIH^[Con]^ and NIH^[1B]^ cells. Shown is the mean ± SD of *n* = 3.

We and others have previously identified the DYRK1B kinase as a negative regulator of Hh signaling in different cell types [14, 24, 25]. In contrast, a recent report described DYRK1B as a positive modulator of the Hh cascade [15], prompting us to reevaluate the role of this kinase in more detail. To this end, we have begun our studies by knocking down endogenous *Dyrk1b* in mouse embryonic fibroblasts stably expressing Sonic Hh ligand (MEF^[SHH]^ cells [31]), which renders these cells constitutively signaling. As can be seen in Figure 1A, an RNAi pool of four different siRNA sequences designed against endogenous *Dyrk1b* led to a significant upregulation of several Hh target genes (*Gli1, Ptch1, Ptch2*) when compared to control siRNA-transfected cells. A de-repression of Hh pathway activity upon *Dyrk1b* knock-down was confirmed by measuring the protein levels of GLI1 (Figure 1A inset). Because DYRK1B had been previously linked to the serum-induced RAS-RAF-MEK pathway [32, 33], which could potentially affect its interaction with Hh signaling, we verified our results using different serum conditions (Figure 1A, 1B). However, using low (0.5%) or high (10%) serum conditions gave almost identical results. Furthermore, testing the four siRNA sequences individually confirmed a de-repression of Hh target gene expression in three out of four cases (Supplementary Figure S1A), arguing for a negative role of endogenous *Dyrk1B* on ligand-induced Hh signaling in fibroblasts. When Hh signaling was blocked by means of a ligand neutralizing antibody (5E1) or by pharmacological SMO inhibition (SANT), *Dyrk1b* knockdown no longer led to increased pathway activity, suggesting that *Dyrk1b* knockdown can modulate active Hh signaling but cannot elicit Hh signaling on its own (Supplementary Figure S1B).

Next, we went on to elucidate the function of this kinase when overexpressed. Therefore, we stably transfected NIH3T3 fibroblasts (a Hh-responsive cell line frequently used in the analysis of the Hh pathway) with an empty control plasmid or with a construct expressing V5-tagged *DYRK1B* (NIH^[Con]^ and NIH^[1B]^ cells; Figure 1C). Treating these cells with the synthetic SMO agonist SAG [34] to stimulate membrane signaling and immunoblotting for the endogenous target gene product GLI1 revealed that the *DYRK1B*-overexpressing cells had lost their SAG-responsiveness (Figure 1D, 1E), arguing that this kinase blocks the signal transmission from SMO to GLI. However, the basal levels of GLI1 were increased in NIH^[1B]^ cells even in the absence of any stimulatory SAG, indicative of a non-canonical activation of GLI activity. Furthermore, we noted that *DYRK1B*-overexpressing cells displayed a GLI1 isoform of about 100 kD in addition to the most abundant 160 kD full-length isoform. Besides this 160 kD variant, control cells possessed a less abundant 130 kD isoform, which was not evident in NIH^[1B]^ cells (Figure 1D). A 100 kD large GLI1 isoform has previously been proposed to represent an inhibitory variant of GLI1 [35]. In agreement with our hypothesis of non-canonical GLI1 activation, we found that the DYRK1B-induced increase in GLI1 levels was largely insensitive to SMO inhibition (Supplementary Figure S1C). Furthermore, the measurement of the mRNA expression levels of Hh target genes (*Gli1, Ptch1*) revealed that *DYRK1B* overexpression blocked SAG-induced Hh signaling while at the same time it increased the basal expression of *Ptch1* (Figure 1F). In contrast, the basal expression of the two major activators of the pathway, *Gli1* and *Gli2*, was drastically reduced in NIH^[1B]^ cells and was close to the technical detection limit, whereas *Gli3* levels were unaffected (Figure 1F and Supplementary Figure S1D). Taken together, our data suggest that DYRK1B inhibits PTCH/SMO-initiated (canonical) Hh signaling while it promotes downstream (non-canonical) activation of the GLI1 transcription factor.

### DYRK1B promotes GLI1 stability

**Figure 2:**
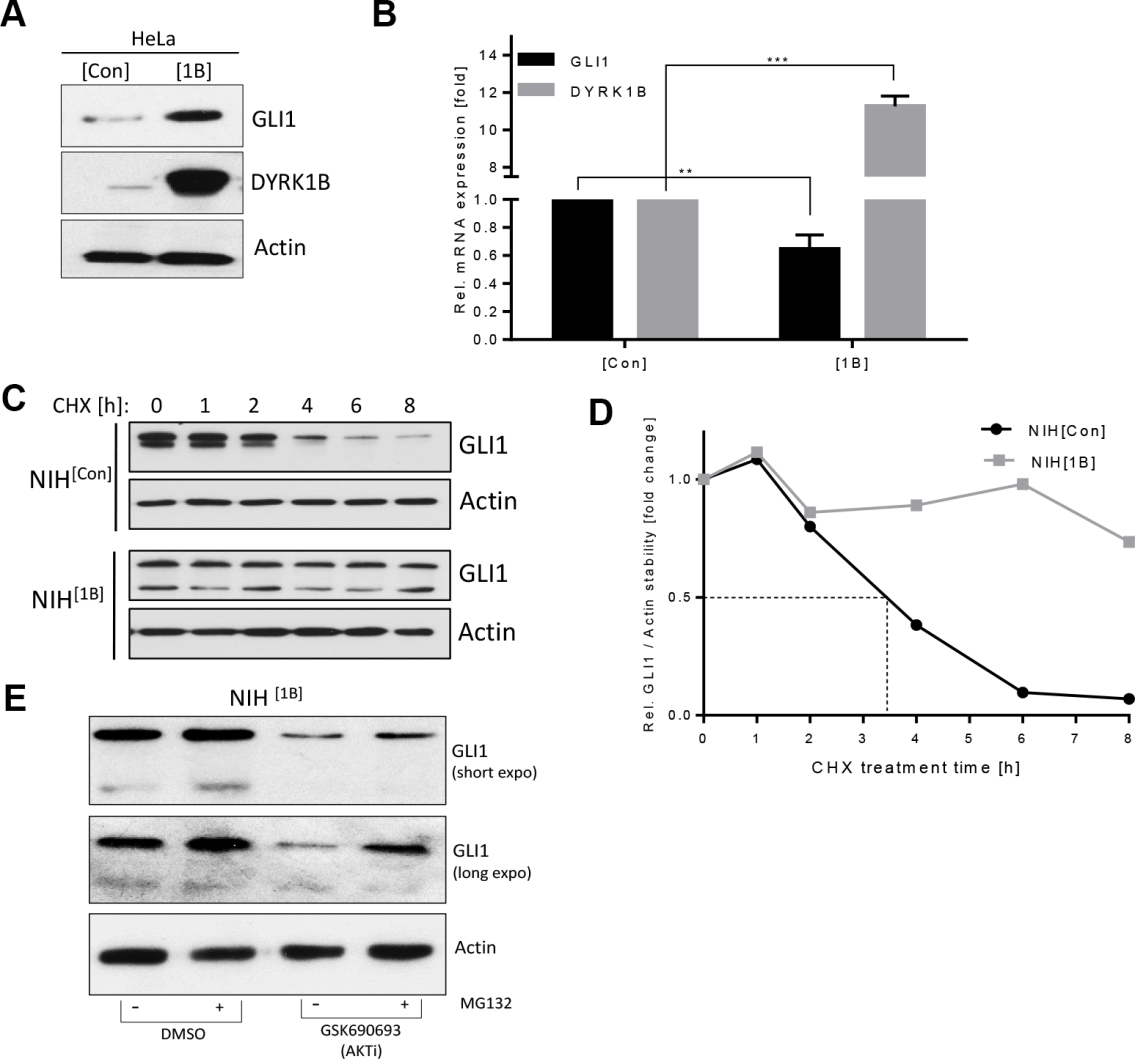
DYRK1B promotes non-canonical GLI1 stabilization (**A**) Detection of GLI1 and DYRK1B by immunoblotting of HeLa cells stably transfected with empty control (mock; HeLa^[Con]^) or *DYRK1B* plasmid (HeLa^[1B]^). (**B**) *GLI1* and *DYRK1B* mRNA expression in HeLa^[Con]^ and HeLa^[1B]^ cells. Shown is the mean ± SD of *n* = 3. (**C**) GLI1 protein stability experiment using Cycloheximide (CHX 100 μg/ml for the indicated times). NIH^[Con]^ and NIH^[1B]^ cells were both pre-treated with 100 nM SAG overnight before addition of Cycloheximide (in continued presence of SAG in order to assure comparability). (**D**) Quantification of the results depicted in (C). Shown is the mean of two independent experiments. (**E**) Levels of endogenous GLI1 in NIH^[1B]^ cells as measured by immunoblotting. Cells were pre-treated with MG132 (20 μM) or DMSO for 1 h, followed by parallel co-treatment with the pan-AKT inhibitor GSK-690693 (10 μM) for 12 h.

We verified the findings made in fibroblasts by overexpressing *DYRK1B* in human cancer cells. In line with our previous observations, stable *DYRK1B* expression in HeLa cells increased the levels of endogenous GLI1 protein (Figure 2A) while at the same time it decreased the *GLI1* mRNA levels (Figure 2B). The fact that GLI1 protein levels were increased upon *DYRK1B* transfection despite its mRNA being decreased argued for a stabilizing effect of DYRK1B on the GLI1 protein. To address this possibility, we performed protein stability assays in NIH^[Con]^ and NIH^[1B]^ cells blocking *de novo* protein synthesis with Cycloheximide. As can be seen in Figure 2C and 2D, endogenous GLI1 was degraded with a half-life (t_1/2_) of approx. 3.5 h in SAG-treated control cells whereas GLI1 protein levels in SAG-treated *DYRK1B*-expressing cells were extremely stable and were only minimally affected over the entire time course of the experiment. Previously, AKT kinase has been shown to promote GLI stability [7, 26, 36]. Therefore, we treated the NIH^[1B]^ cells, which possess stabilized GLI1 (Figure 2C, 2D), with a pan-AKT inhibitor (GSK-690693). In line with a GLI-stabilizing role of AKT also in these cells, GLI1 levels dropped significantly upon AKT inhibition, an effect which could be rescued by pharmacological blockade of the proteasome (Figure 2E). In summary, we could show that ectopically expressed DYRK1B kinase promotes an increase in GLI1 protein stability and that this effect is likely mediated through AKT.

### DYRK1B activates the PI3K/mTOR/AKT pathway

**Figure 3:**
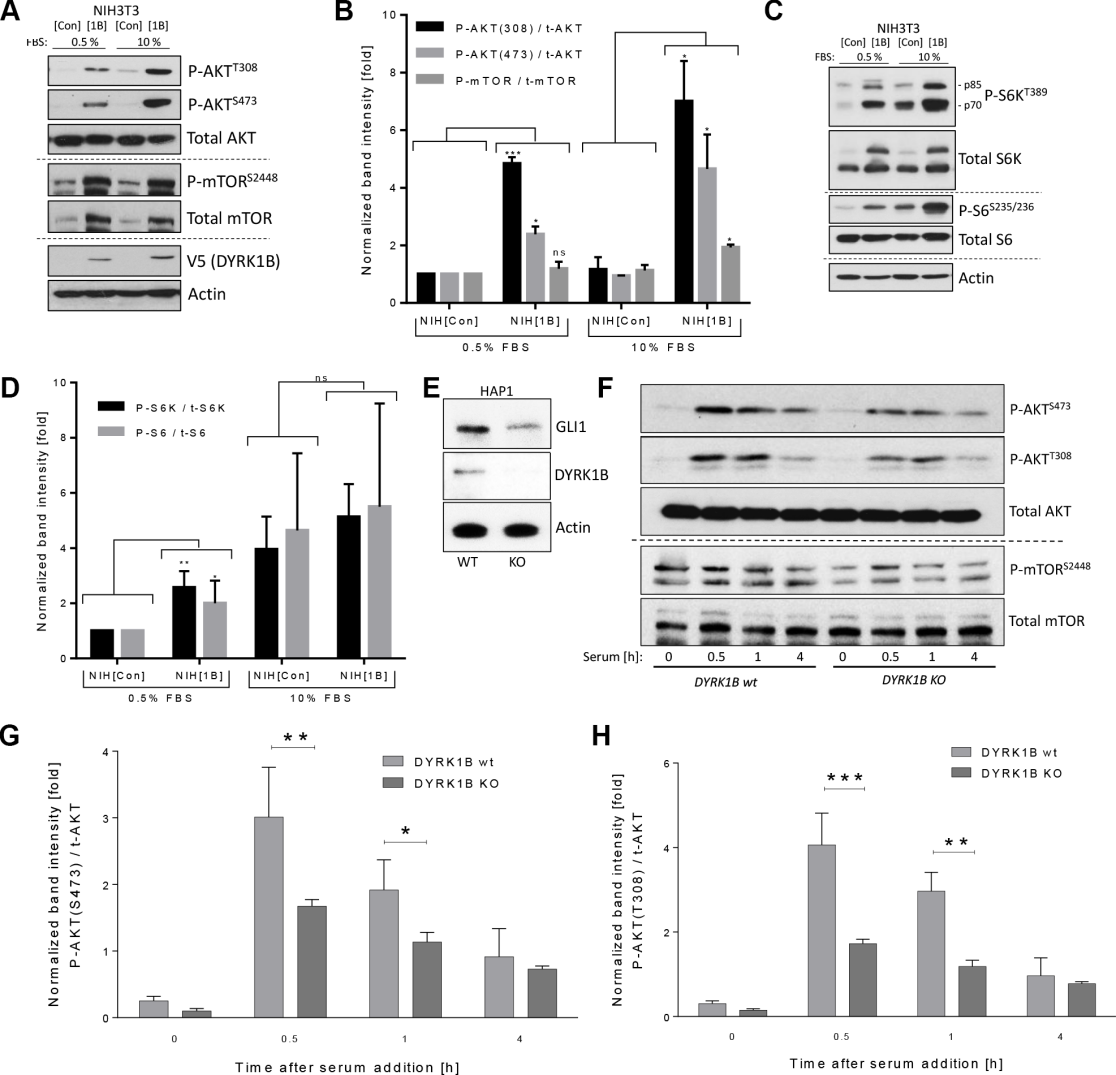
DYRK1B activates the mTOR/AKT pathway (**A**) Detection of AKT and mTOR proteins by immunoblotting of NIH^[Con]^ and NIH^[1B]^ cell lysates. (**B**) Quantification of the results depicted in (A). Shown are band intensities of the phosphorylated proteins (e.g. P-AKT(308)) normalized to the respective total protein (e.g. t-AKT); mean ± SD of *n* = 3. (**C**) Detection of S6-Kinase (S6K) and S6 proteins by immunoblotting of NIH^[Con]^ and NIH^[1B]^ cell lysates. (**D**) Quantification of the results depicted in (C). Shown are band intensities of the phosphorylated protein normalized to the respective total protein; mean ± SD of *n* = 3. (**E**) Western blot using lysates from wildtype (wt) and from *DYRK1B*-knock out (KO) HAP1 cells. (**F**) Kinetics of AKT and mTOR phosphorylation in *DYRK1B* wt and KO HAP1 cells. Cells were starved in 0% FBS-containing media for 24 h before addition of 10% serum (FBS) for the indicated times. (**G**) Quantification of AKT^Ser473^ phosphorylation as depicted in (F). Shown is the mean ± SD of *n* = 3. (**H**) Quantification of AKT^Thr308^ phosphorylation as depicted in (F). Shown is the mean ± SD of *n* = 3.

Since our data suggested that AKT might play a role in the GLI1-stabilizing impact of DYRK1B, we analyzed the levels of activated (phosphorylated) AKT and mTOR. Interestingly, an induction of phosphorylation on mTOR-Ser2448 and on AKT-Ser473 and Thr308 could be observed in response to elevated *DYRK1B* expression (Figure 3A, 3B). As AKT^Ser473^ is exclusively phosphorylated by mTORC2 [29, 30], these data suggest that DYRK1B directly or indirectly activates this multi-protein complex. In addition, the PDK1-induced phosphorylation of Thr308 in AKT was also increased, suggesting that DYRK1B might activate the entire PI3K/mTORC2/AKT signaling arm or that both phospho-sites communicate and influence each other. In order to investigate whether the second mTOR complex (mTORC1) was also activated, we measured the phosphorylation status of one of its major targets, S6-Kinase (S6K-Thr389) and the S6K target ribosomal protein S6 (S6-Ser235/Ser236). In agreement with the induction of mTORC2/AKT activity, also the mTORC1 complex was activated by DYRK1B, as evidenced by stimulated phosphorylation of S6K and S6. However, this effect was less evident under high serum conditions, when basal levels of phospho-S6K and phospho-S6 are quite high (Figure 3C, 3D). In order to verify that DYRK1B overexpression also induces phosphorylation of PI3K/AKT pathway members in human cells, we analyzed stably *DYRK1B*-expressing HeLa cells (Figure 2A) for AKT and mTOR phosphorylation. In line with our data obtained in fibroblasts, *DYRK1B*-expressing cells displayed elevated levels of mTOR^S2448^ and AKT^S473/T308^ phosphorylation (Supplementary Figure S2A).

Next, we were eager to investigate the effects on mTOR/AKT in cells genetically depleted of *DYRK1B* through CRISPR/Cas9 methodology (Figure 3E). Here, we made use of mammalian HAP1 cells harboring a haploid genome, which facilitates the efficacy of CRISPR-based approaches [37]. Interestingly, *DYRK1B*-knock out (KO) cells displayed reduced endogenous GLI1 protein levels (Figure 3E). Furthermore and in congruence with our previous findings, serum-stimulated *DYRK1B*-KO cells demonstrated an overall reduced level of AKT (Ser473; Thr308) and mTOR (Ser2448) phosphorylation (Figure 3F–3H and Supplementary Figure S3A). Furthermore, in line with mTOR favoring cell growth and proliferation [28], *DYRK1B*-KO cells proliferated significantly slower than the parental *DYRK1B* wildtype cells (Supplementary Figure S3B). Taken together, our data imply that DYRK1B is an activator of the PI3K/mTOR/AKT signaling pathway.

### DYRK1B contributes to Hh-induced mTOR/AKT activation

**Figure 4:**
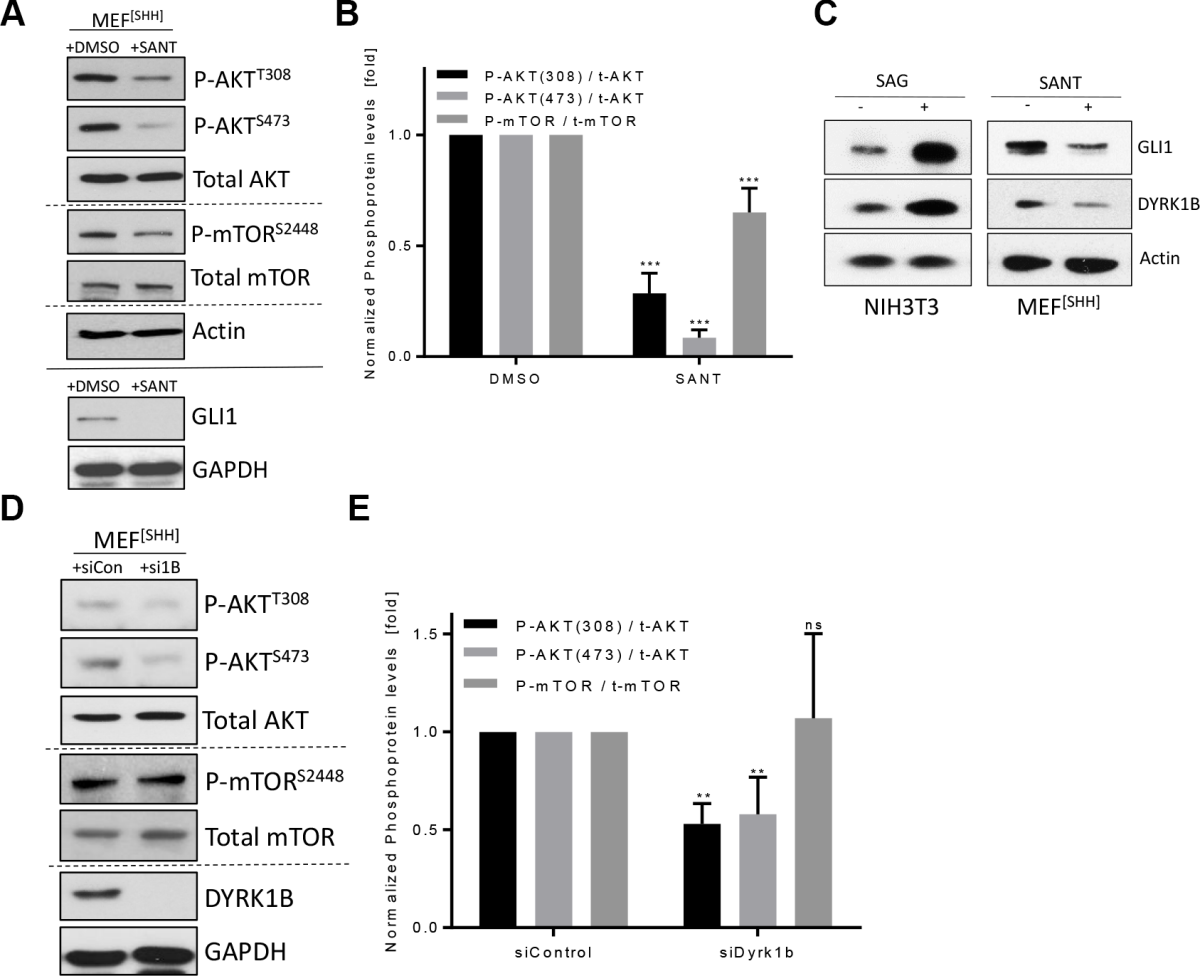
DYRK1B contributes to Hh-induced phosphorylation reactions (**A**) Detection of AKT and mTOR proteins by immunoblotting of MEF^[SHH]^ cell lysates. Cells were treated with DMSO or SANT (0.2 μM) for 2–3 d before lysis. (**B**) Quantification of the results shown in (A). Shown is the mean ± SD of *n* = 3. (**C**) Levels of endogenous GLI1 and DYRK1B protein as measured by Western blotting of NIH3T3 or MEF^[SHH]^ cell lysates. Treatment (48 h) as indicated. (**D**) Detection of AKT and mTOR proteins by immunoblotting of MEF^[SHH]^ cell lysates after transfection with control siRNA (siCon) or with a pool of four different *Dyrk1b*-specific RNAi sequences (si1B). (**E**) Quantification of the results shown in (D). Shown is the mean ± SD of *n* = 3.

Continuing from these observations, we went on to elucidate the link between these phosphorylation events and Hh signaling. First we verified literature data that fibroblasts respond to Hh pathway stimulation with activation of AKT and mTOR (Supplementary Figure S4A). Similarly, continuously signaling MEF^[SHH]^ cells displayed elevated levels of phosphorylated AKT and mTOR, which could be suppressed by inhibition of the Hh pathway with the SMO antagonist SANT (Figure 4A, 4B) [34]. As our previous data suggested that DYRK1B activates the same set of kinases, we wondered whether DYRK1B was involved in mediating the effect of Hh signaling on AKT/mTOR. Indeed, stimulating the Hh pathway led to an elevation of DYRK1B protein levels and, conversely, suppressing active Hh signaling reduced DYRK1B levels in two different fibroblast cell lines (Figure 4C). We therefore went on to analyze phospho-mTOR/AKT levels in MEF^[SHH]^ cells after *Dyrk1b* knock-down. As can be seen in Figure 4D and 4E, the levels of phospho-AKT^Ser473^ and phospho-AKT^Thr308^ were significantly reduced upon knock-down of *Dyrk1b*. The levels of phospho-mTOR remained constant, which was surprising in light of the reduced AKT^Ser473^ (a selective mTORC2 substrate) levels after *Dyrk1B* knock-down. However, as the antibody detects the entire mTOR pool, this might reflect a differential effect of endogenous DYRK1B on mTORC2 versus mTORC1. Supporting our data of reduced Hh-induced AKT (mTOR) phosphorylation in the absence of DYRK1B in MEF cells, we could obtain similar results using NIH3T3 cells (Supplementary Figure S4B). In summary, our data suggest that Hh signaling induces the phosphorylation of AKT (and potentially mTOR) through DYRK1B.

### The kinetics of DYRK1B-mediated GLI regulation

**Figure 5:**
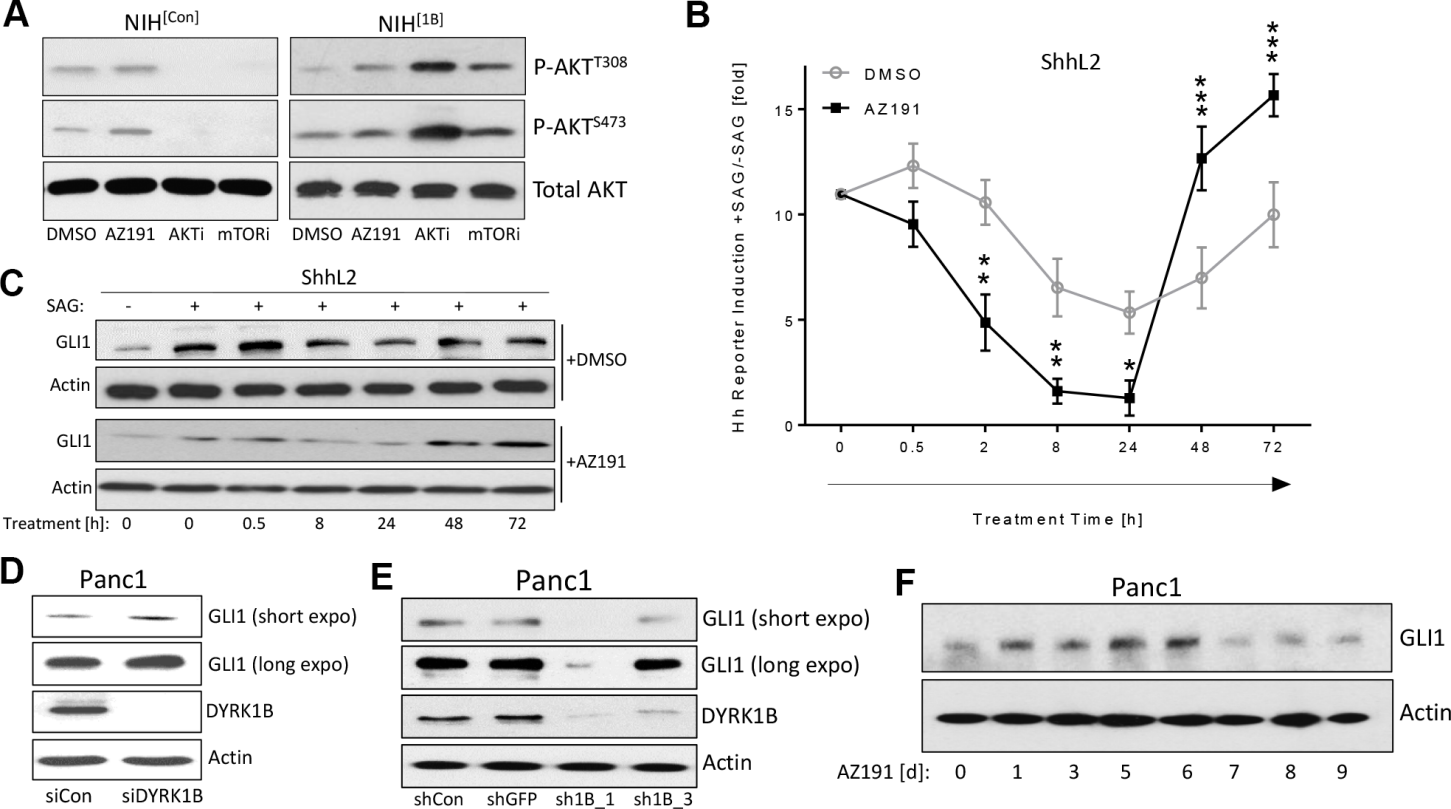
Kinetics of DYRK1B-induced effects (**A**) Detection of AKT phosphorylation in NIH^[Con]^ and NIH^[1B]^ cells. Treatment was in 0.5% FBS for 24 h. DYRK1B-inhibitor (AZ191; 1 μM); AKT-inhibitor (AKTi: GSK-690693; 10 μM); mTOR-inhibitor (mTORi: KU-0063794; 1 μM). (**B**) Time course experiment in ShhL2 cells (Hh reporter cells). Cells were plated and 24 h later, SAG (100 nM) was added for another 24 h. Then, DMSO or AZ191 (1 μM) was added to the wells (keeping SAG as well) for the indicated treatment times. The statistical significances were calculated comparing DMSO vs AZ191-treated samples. Shown is the mean ± SD of *n* = 3. (**C**) GLI1 Western blot of ShhL2 lysates as shown in (A). (**D**) GLI1 protein levels in Panc1 cells transfected with control siRNA (*siCon*) or *DYRK1B*-specific siRNA pool (*siDYRK1B*). Cells were harvested 3d after transfection. (**E**) GLI1 protein levels in Panc1 cells transfected with control shRNA plasmids (*shCon, shGFP*) or *DYRK1B*-specific shRNA plasmids (*sh1B_1, sh1B_3*). Cells were harvested 7d after transfection. (**F**) Levels of endogenous GLI1 in Panc1 cells treated for the indicated times with 1 μM of AZ191.

Our data thus far implied that DYRK1B stimulates the mTOR/AKT pathway, which subsequently promotes GLI stabilization. The PI3K/mTOR/AKT system is subject to intense feed-back regulation, resulting in e.g. pronounced upregulation of phospho-AKT in the case of mTORC1 inhibitors, which has also created difficulties with the clinical use of this compound class [27, 28]. We were therefore interested to see how the Hh pathway would be regulated over time after DYRK1B inhibition. First, we investigated the systemic feedback regulation by analyzing AKT phosphorylation in NIH^[MCS]^ and NIH^[1B]^ cells upon inhibition of AKT (GSK-690693, a pan-AKT inhibitor), mTOR (KU-0063794, a dual mTORC1/2 inhibitor) and DYRK1B (AZ191, a selective small molecule DYRK1B inhibitor [38]). As can be seen in Figure 5A, all inhibitors led to a subsequent increase in phospho-AKT levels in *DYRK1B*-overexpressing cells, although they were different in amplitude. In contrast, in wildtype NIH^[MCS]^ cells, AKT and mTOR inhibition resulted in reduced phospho-AKT levels while AZ191 led to an increase. Taken together with the previous experiments, this result strongly suggested that DYRK1B is indeed involved in a complex regulatory mTOR/AKT feedback loop.

Next, we turned to ShhL2 cells (a clonal NIH3T3 cell line harboring a Hh/GLI-responsive luciferase reporter construct in the genome [39]) and pre-treated these cells with SAG to activate Hh signaling. Then, AZ191 was added for different time periods and the activity of Hh signaling was recorded. As can be seen in Figure 5B and 5C, when compared to the DMSO control, Hh signaling was suppressed by AZ191 in the first 24 h and was then increased over controls at later time points (48–72 h), suggesting a pronounced influence of feedback regulation on the kinetics of the Hh response. These data show that the exact time point of analysis is important when determining the effects of DYRK1B.

In order to analyze the issue of kinetics further, we knocked down endogenous *DYRK1B* in human Panc1 pancreatic cancer cells by two different approaches: 1.) In a short-term experiment (2–3 d), short-interfering RNA (siRNA) was used and 2.) In a long-term experiment (6–7 d), short hairpin RNA (shRNA) was applied. The acute knock-down of *DYRK1B* by means of siRNA (short-term) resulted in an increase of endogenous GLI1 levels (Figure 5D and Supplementary Figure S5A). In contrast, the long-term knock-down of *DYRK1B* through a shRNA approach (Supplementary Figure S5B, S5C) led to a suppression of GLI1 expression (Figure 5E and Supplementary Figure S5D, S5E) and reduced levels of phosphorylated AKT and mTOR (Supplementary Figure S5F). To corroborate these findings and to rule out potential effects of siRNA versus shRNA technology, we performed a time course experiment treating Panc1 cells for 9 d with AZ191 and determined the daily changes in GLI1 levels (Figure 5F and Supplementary Figure S5G). In line with our previous findings, GLI1 protein levels undulated during this time frame and were induced during the first 6 days, followed by a reduction below the DMSO control levels afterwards (7–9 d). In summary and in agreement with DYRK1B impinging on the strongly feedback-regulated mTOR/AKT kinase system, we could observe a prominent time-dependent impact of DYRK1B inhibition on GLI1 levels.

### Targeting DYRK1B in GLI-dependent cancer cells

**Figure 6:**
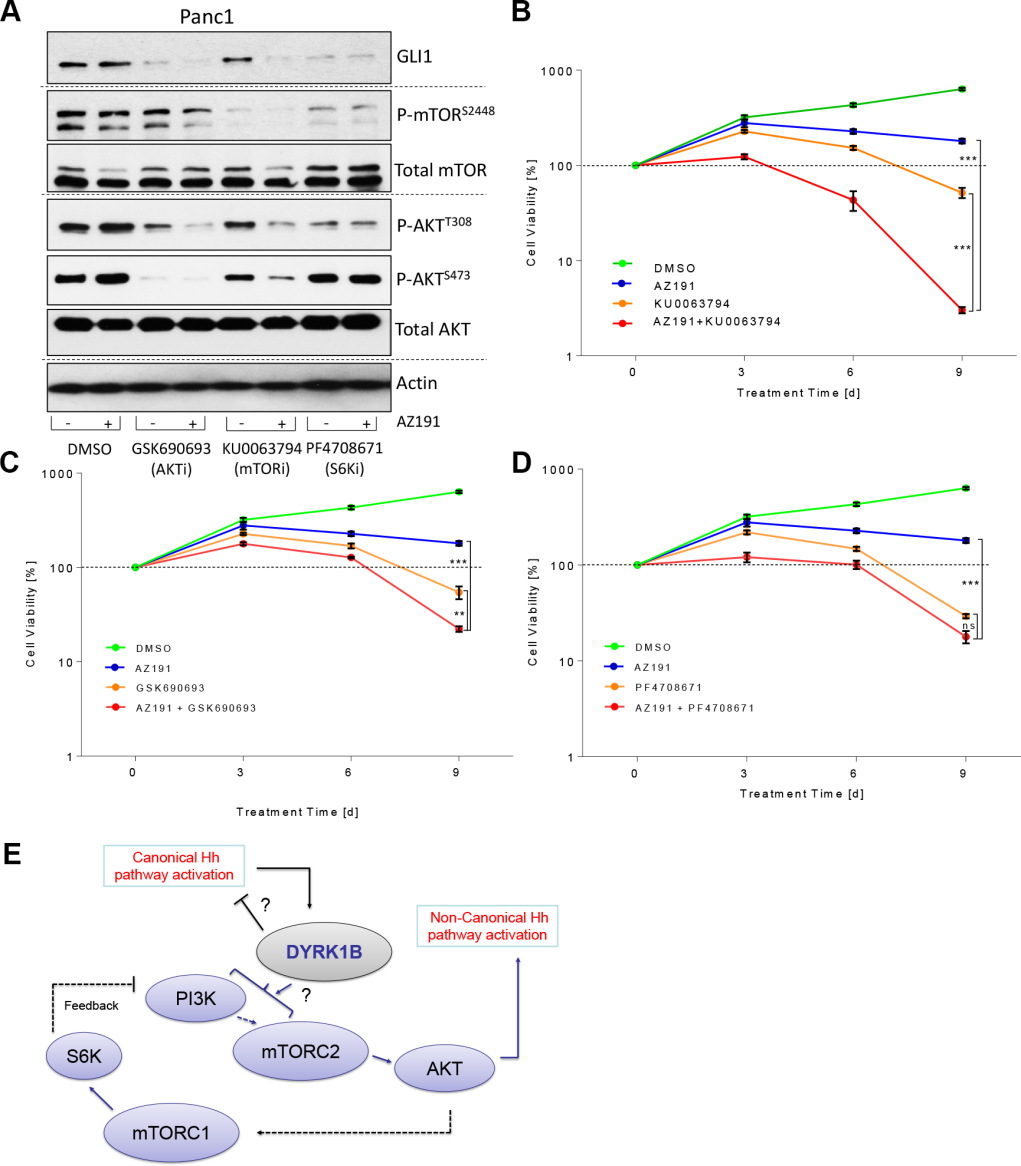
Improving the therapeutic targeting of DYRK1B (**A**) Immunoblot showing the levels of GLI1, AKT and mTOR proteins in Panc1 cells upon treatment (24 h, 0.5% FBS) with various inhibitors. GSK-690693 (pan-AKT inhibitor): 10 μM; KU-0063794 (dual mTORC1/2 inhibitor): 1 μM; PF-4708671 (S6K1 inhibitor): 10 μM. (**B**) Panc1 growth curve (mean ± SD of *n* = 3). Cells were treated with DMSO, AZ191 (1 μM), KU-0063794 (dual mTOR inhibitor; 1 μM) or combinations as indicated. (**C**) Panc1 growth curve (mean ± SD of *n* = 3). Cells were treated with DMSO, AZ191 (1 μM), GSK-690693 (pan-AKT inhibitor; 10 μM) or combinations as indicated. (**D**) Panc1 growth curve (mean ± SD of *n* = 3). Cells were treated with DMSO, AZ191 (1 μM), PF-4708671 (S6K1 inhibitor; 10 μM) or combinations as indicated. (**E**) Graphical depiction of our findings on DYRK1B-mediated regulation of Hh pathway activity. Solid and punctate lines depict direct and indirect interactions, respectively. The exact mechanism of PI3K/mTORC2 activation by DYRK1B requires further investigation.

From a therapeutic point of view, the fluctuating kinetics of GLI1 levels following a DYRK1B inhibition are problematic as suboptimal or short-term treatments with DYRK1B antagonists might result in concomitant upregulation of oncogenic GLI1 in cancer cells. This might be particularly true if these cells express high levels of *DYRK1B*, such as many pancreatic and ovarian cancer cells. We therefore tested the combination of AZ191 (DYRK1B inhibitor) with drugs targeting mTORC1/2 (KU-0063794), AKT (GSK-690693) or S6K (PF-4708671) and measured the effects on GLI1 levels in *DYRK1B*-amplified Panc1 cells (Figure 6A and Supplementary Figure S6A–S6D). Treatment with AZ191 alone (24 h) increased the phosphorylation of AKT and the GLI1 expression, whereas co-treatment with the mTOR/AKT/S6K inhibitors significantly reduced the levels of both. The effect was most clearly seen with the dual mTORC1/2 inhibitor (KU-0063794), which on its own had little effect on GLI1. In combination with AZ191 however, GLI1 levels were almost completely abrogated (Figure 6A and Supplementary Figure S6A–S6D). As Panc1 cells grow in a GLI1-dependent manner [13, 15, 40], we next turned to cell growth assays to measure the cytotoxic impact of these inhibitors. While single treatment with AZ191 or KU-0063794 alone displayed only a moderate effect on cell growth (cytostatic), the combination of both drugs was strongly cytotoxic to pancreatic cancer cells (Figure 6B). Comparable results were obtained with the combination treatment of AZ191 plus AKT inhibitor (Figure 6C). The S6K antagonist was quite effective as monotherapy and a significant additional effect of AZ191 was therefore not possible to detect (Figure 6D). Intriguingly, knock-down of *GLI1* significantly reduced the cell growth retardation seen with the various inhibitors, highlighting the importance of GLI1 in mediating many of the observed anti-proliferative effects (Supplementary Figure S7A–S7E). Moreover, the results on dual DYRK1B-PI3K/AKT/mTOR/S6K inhibition were not specific to Panc1 cells as we could reproduce them in Ovcar-3 ovarian cancer cells using combinations of AZ191 and inhibitors targeting PI3K, mTOR, AKT and S6K (Supplementary Figure S8A–S8E).

Taken together, we propose that a dual targeting approach combining a DYRK1B antagonist with an inhibitor of the PI3K/mTOR/AKT pathway has a pronounced impact on the GLI1 oncoprotein and exerts strong cytotoxic effects in cancer cells.

## DISCUSSION

Previous findings on the role of DYRK1B in the Hh pathway were inconclusive and positive [15] as well as negative [14, 24, 25] regulatory functions were ascribed to this kinase. Here, we attempted to bring together these differing results and clarify the role of DYRK1B in more detail. Our data reveal a complex interaction of this kinase with mammalian Hh/GLI regulation showing dual and sometimes opposing effects: 1.) The ectopic expression of *DYRK1B* potently blocked canonical SMO-initiated signaling. The underlying mechanism of this negative regulation requires further investigations. 2.) In contrast, overexpressed *DYRK1B* enhanced the protein stability of GLI1 by preventing its proteasomal degradation. This stabilizing effect is most likely executed through AKT, which we found to be activated by DYRK1B and which is known to phosphorylate and protect GLI transcription factors from decay [7, 26]. The exact mechanism of AKT stimulation by DYRK1B is currently unknown and requires future work. 3.) Because of DYRK1B's ability to activate the PI3K/mTOR/AKT pathway, the whole DYRK1B-Hh/GLI-system is subject to pronounced feedback control, resulting in a strong influence of kinetics on the actual Hh pathway output. Therefore, short-term inhibition of DYRK1B resulted in an enhancement of Hh signaling whereas long term blockade of DYRK1B function was associated with suppression of GLI1 levels. We believe that these findings can explain many, if not all, published effects of DYRK1B on Hh/GLI signaling and suggest that most previous studies might represent only one specific aspect of the entire crosstalk spectrum. A comparable controversy attributes to the role of oncogenic RAS on Hh signaling [11, 14, 41, 42] and it is interesting to note that DYRK1B has been described as a downstream effector of mutant KRAS [32]. Moreover, our connection presented here between DYRK1B and PI3K/mTOR/AKT signaling might explain why DYRK1B was discovered in a large screen identifying synthetic lethal gene partners of mutant *KRAS* [43]. In addition, its involvement in PI3K signaling and the discovery of *DYRK1B* mutations in the metabolic syndrome are intriguing [25, 44, 45].

Strikingly, we could observe that, at least in one specific cell line tested, the stress-induced DYRK1B kinase was able to potently stimulate GLI1 protein stability even in the absence of clearly measurable *Gli1* and *Gli2* mRNA expression (Figure 1F, Supplementary Figure S1D). Thinking about possible routes of GLI1 activation in several pathological situations, it is tempting to speculate about Hh target gene induction being potentially triggered by completely Hh-unrelated regulators inducing *DYRK1B* expression, bypassing the need for increased *GLI1/2* mRNA levels. Moreover, in one cell line, ectopic *DYRK1B* expression altered the appearance of GLI1 protein isoforms and promoted the generation of a shorter variant of about 100 kD. Although a variant of this size has been suggested to be inhibitory [35], another shorter isoform of 130 kD seems to be activating [46]. How DYRK1B is generating this shorter GLI1 isoform, how general the effect is and what the role of this shorter GLI1 variant might be warrants further investigations.

It is well established that Hedgehog signaling induces numerous kinases including AKT, but the underlying mechanism has been elusive. Here, we could demonstrate that SMO activation results in upregulation of DYRK1B and that depletion of this kinase by means of RNAi abrogates the ability of Hh signaling to stimulate AKT phosphorylation. As this also applies to Ser473 phosphorylation of AKT, a faithful read-out of mTORC2 functionality [29, 30], we hypothesize that DYRK1B might be involved in mTORC2 regulation. In line with this assumption, we found that siRNA against *Dyrk1b* reduced the levels of phospho-AKT^Ser473^ (marker for mTORC2 activity), but had little repressive effect on phospho-S6/S6K (read-out of mTORC1 activity) (not shown). With respect to the activation of the pro-survival kinase AKT, it is interesting to note that DYRK1B has actually been described as a survival kinase before [21, 47].

In summary, we could describe a surprisingly complex crosstalk between DYRK1B and Hh signaling. According to our model, the exact net result of DYRK1B's impact on the Hh pathway might be dependent on DYRK1B expression level, canonical/non-canonical Hh signaling, time point of analysis and/or cell type. In a clinical situation aiming to target the DYRK1B survival kinase, considering all these different aspects will be impossible. Therefore, we have tested a combination treatment targeting DYRK1B and the mTOR/AKT pathway in a proof-of-principle study. Using *DYRK1B*-amplified pancreatic and ovarian cancer cells, co-targeting both kinases resulted in a significantly reduced GLI1 level and in increased cell death induction which could help to design new cancer therapies in the future.

## MATERIALS AND METHODS

### Reagents

Smoothened agonist SAG was purchased from Calbiochem. SANT (SANT-1); KU-0063794, GSK-690693, PF-4708671 and MG132 were from Sigma and AZ191 was from SelleckChem. Cycloheximide was purchased from Biomol.

The monoclonal Hedgehog neutralizing antibody (5E1, supernatant), developed/deposited by T.M. Jessell/S. Brenner-Morton was obtained from the Developmental Studies Hybridoma Bank, created by the NICHD of the NIH and maintained at The University of Iowa, Department of Biology, Iowa City, IA 52242.

### Cell lines

NIH3T3, HeLa, ShhL2, Panc1, OvCar3 cell lines were purchased from ATCC. All cell lines were cultured in Dulbecco's Modified Eagle Medium (DMEM (high Glucose plus Glutamine and Pyruvate), Invitrogen) supplemented with 10% fetal bovine serum (FBS) and 1% Penicillin/Streptomycin at 37°C with 5% CO_2_. MEF^[SHH]^ cells were kindly provided by Wade Bushman [31]. *DYRK1B* wildtype and knock-out haploid HAP1 cells (#HZGHC000363c010) were purchased from Horizon Genomics (Vienna, Austria). If not otherwise stated, serum concentrations were reduced to 0.5% during experiments for all cell types.

### Generation of stable cells

The coding sequence of human *DYRK1B* (isoform a) was PCR amplified and cloned into pEF6/V5-His using TOPO cloning (Invitrogen) yielding pEF-DYRK1B. NIH3T3 and HeLa cells were transfected with empty vector or with pEF-DYRK1B and cell clones surviving Blasticidin selection were pooled.

### Luciferase reporter assays

ShhL2 cells were plated in triplicate and were grown to full confluence in solid white 96-well plates with clear bottom. Subsequently, cells were treated in 5% FBS-containing medium with 100 nM SAG plus the respective compounds for the indicated times. Cells were lysed in Passive Lysis Buffer (Promega) and Firefly and Renilla Luciferase activity were measured using an Orion L microplate luminometer (Berthold Detection Systems) using Beetle- and Renilla-Juice reagents (both PJK).

### Immunoblotting

Separation of lysates by SDS-PAGE was followed by subsequent Western Blot analysis. SDS-PAGE gels were blotted on Immobilon-PVDF membranes (Millipore) and incubated with the respective primary antibody, followed by an HRP-coupled secondary antibody. Detection of the HRP signal was performed using Pierce ECL Western Blotting Substrate (Thermo Scientific) according to the manufacturer's protocol. The following primary antibodies were used: α-DYRK1B (#5672; Cell Signaling Technology (CST)); α-GLI1 (#2643; CST); α-total AKT (#9272; CST); α-phospho-AKT^Ser473^ (#9271; CST); α-phospho-AKT^Thr308^ (#13038; CST); α-phospho-mTOR^Ser2448^ (#5536; CST); α-total mTOR (#2983; CST); α-V5 (#R960-25; Invitrogen); α-GAPDH (#G9545; Sigma); α-Actin (#A5441; Sigma).

### Small-interfering RNA (siRNA) and short hairpin RNA (shRNA) transfection

Cells were transfected with 35 nM siRNA (Dharmacon SMARTpools and Qiagen control siRNA using RNAiMax (Invitrogen). Control siRNA (siCon) was purchased from Qiagen (All-Stars-siRNA; siAll). The *Dyrk1b*-specific siRNA target sequences were: si1B_1: AUA CAGAGAUGAAGUACUA; si1B_2: GCACAUCAAU GAGGUAUAC; si1B_3: GAGAUGAAGUACUACAU AG; si1B_4: GGACAAAGGAACUCAGGAA: The mouse *Gli2*-specific and the human *DYRK1B*-specific siRNAs have been described before [14, 48].

Short-hairpin RNA (shRNA) target sequences in pLKO. 1-puro backbone (Mission, obtained through Sigma) were as follows: shCon (SHC002; scrambled control): CAACAAGATGAAGAGCACCAA; shGFP (SHC005; targeting EGFP): TACAACAGCCACAACGT CTAT; shDYRK1B_1: GACCTACAAGCACATCAAT GA; shDYRK1B_3: CACGGAGATGAAGTACTATAT; shGLI1_1: CATCCATCACAGATCGCATTT. Panc1 cells were transfected on day 0 in 10% FBS media. Medium was changed to 0.5% FBS media and Puromycin (2 μg/ml) was added on day 1 (cells were kept like this until day 6). After recovery for 1 d in medium without selective pressure, cells were harvested on day 7 (for shDYRK1B) or day 9 (for shGLI1).

### RNA preparation, cDNA synthesis, qPCR

Total RNA was extracted using NucleoSpin RNA II kit (Macherey-Nagel) according to the manufacturer's protocol. cDNA synthesis of 1 μg total RNA was performed using iScript cDNA Synthesis Kit (Biorad) following the manufacturer's guidelines. Quantitative PCR reactions were performed using the Absolute QPCR SYBR Green Mix (ABGene). qPCR reactions were performed on 96 well qPCR plates (ABGene) using either the Mx3000P or Mx3005P qPCR systems (Agilent). Results were calculated as relative mRNA expression (2^ΔΔCt^). Data was obtained from at least three independent experiments and is shown as the mean ± StDev. Primer sequences (5′ to 3′) for the detection of mouse *Dyrk1b* were: For-TTGACACCTGCCCCTCCTCTAGCAC; Rev-GGCCC CCACAATATCGGTTGCTGTA. Human *DYRK1B*: For-T TGGCCAGGTGGTGAAAGCCTATGA; Rev-CAATCTG GGCCTGGTTCAGGAAAGC. All other primer sequences have been described elsewhere [13, 48–50].

### Statistical analysis

Unless otherwise stated, data is presented as the mean of three independent experiments ± standard deviation (StDev). Statistical significance was calculated by applying a two-tailed student's *t-test*. *p < 0.05; **p < 0.01; ***p < 0.001.

## SUPPLEMENTARY MATERIALS


