# Supplementary material for: DYRK1B blocks canonical and promotes non-canonical Hedgehog signaling through activation of the mTOR/AKT pathway
Source: Oncotarget. 2016 Nov 26;8(1):833–45. doi: 10.18632/oncotarget.13662 (PMC5352201; doi:10.18632/oncotarget.13662)
Supplement: Supplementary file 1 [file oncotarget-08-833-s001.pdf]

DYRK1B blocks canonical and promotes non-canonical hedgehog signaling through activation of the mTOR/AKT pathway

Supplementary Materials

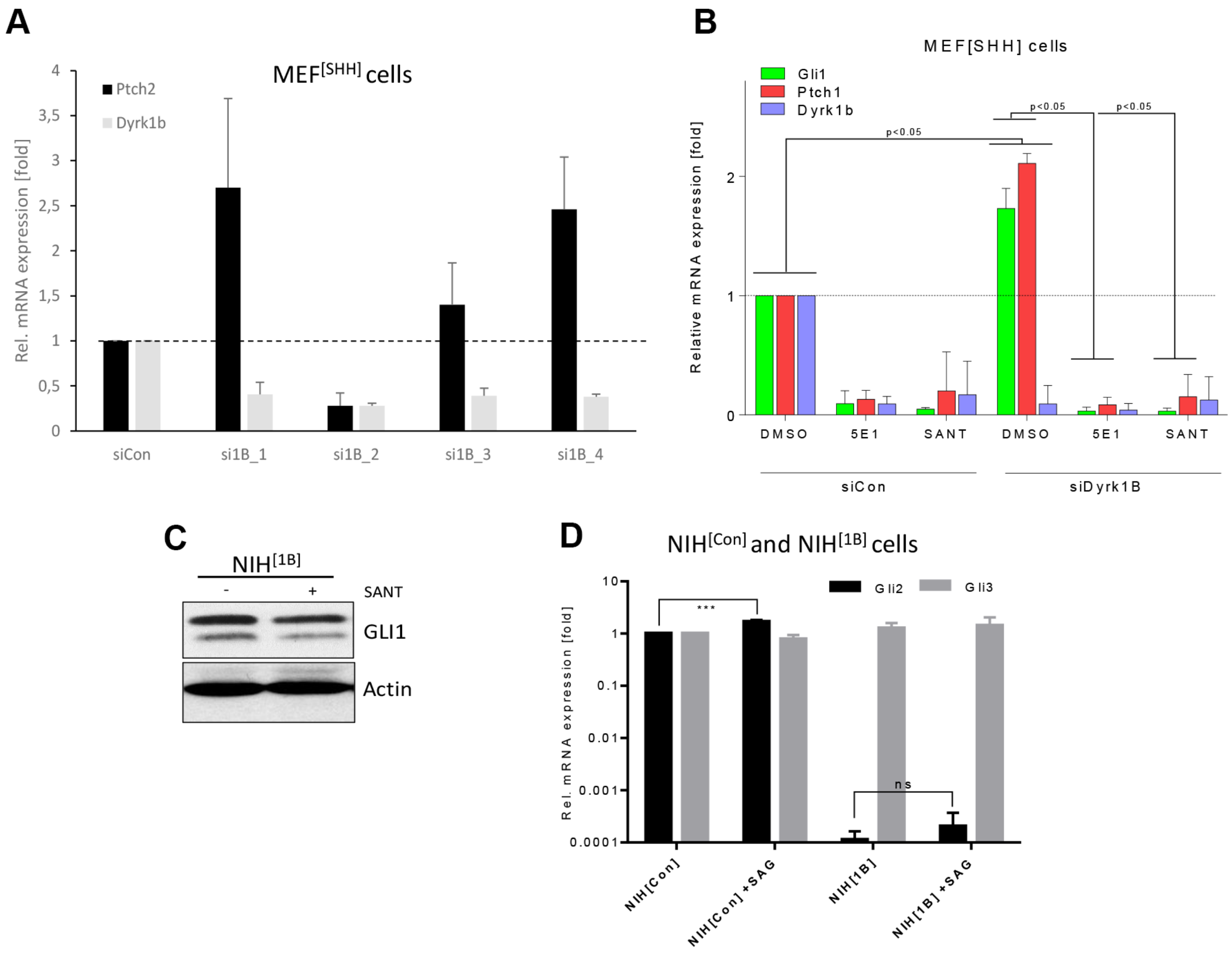

Supplementary Figure S1: Opposing effects of DYRK1B on Hh signaling. (**A**) Detection of *Dyrk1b* and the Hh target gene *Ptch2* and by means of qPCR. MEF[SHH] cells were transfected with control siRNA (siCon) or four individual RNAi sequences targeting *Dyrk1b*. (**B**) MEF[SHH] cells were transfected with control or *Dyrk1b*-specific siRNA and were subsequently treated with either DMSO, SANT (0.2 µM) or the Hh ligand neutralizing antibody 5E1 (2.5 µg/ml) for 48 h in 0.5% FBS. *Gli1*, *Ptch1* and *Dyrk1b* mRNA transcripts were measured by qPCR and the statistical significance of selected comparisons is given. (**C**) GLI1 protein levels in NIH[1B] treated with DMSO or SANT (0.2 µM) for 48 h (0.5% FBS). (**D**) *Gli2* and *Gli3* mRNA levels in NIH[Con] and NIH[1B] cells treated with SAG (100 nM) for 48 h.

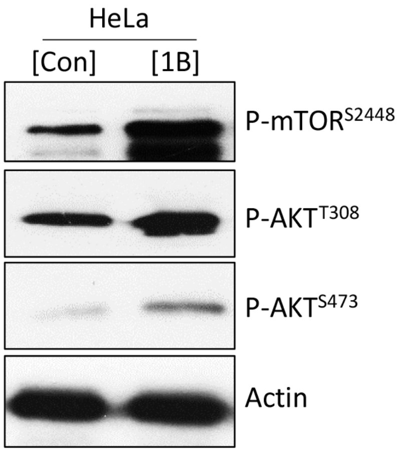

Supplementary Figure S2: DYRK1B activates the PI3K/mTOR/AKT pathway in human cells. (**A**) Western blot showing AKT and mTOR phosphorylation in control Hela$^{[Con]}$ (stably transfected with empty vector control) and in stably *DYRK1B*-overexpressing Hela$^{[1B]}$ cells (this panel relates to Figure 2A).

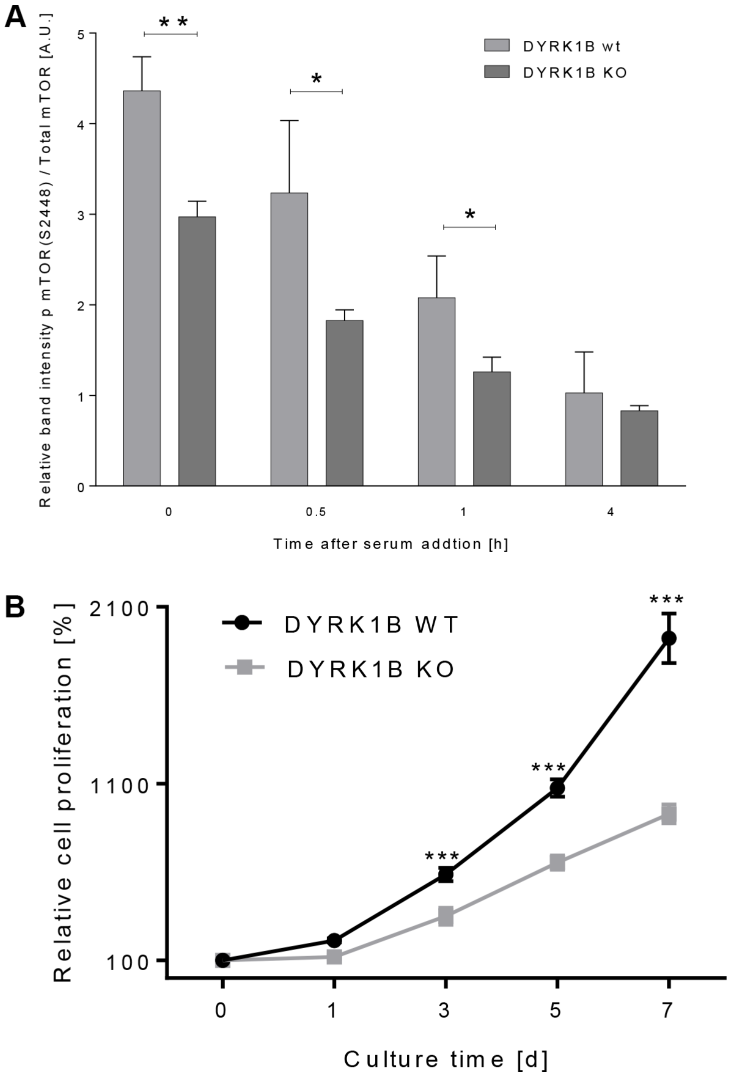

Supplementary Figure S3: DYRK1B affects the PI3K/mTOR/AKT pathway. (**A**) Quantification of phospho-mTOR levels in serum-stimulated *DYRK1B* wildtype (wt) and knock out (KO) HAP1 cells as depicted in Figure 3F. Shown is the mean ± SD of $n = 3$. (**B**) Growth curve of *DYRK1B* wt and KO HAP1 cells in 0.5% FBS-containing media over seven days. Significances are calculated between the corresponding time points of wt and KO.

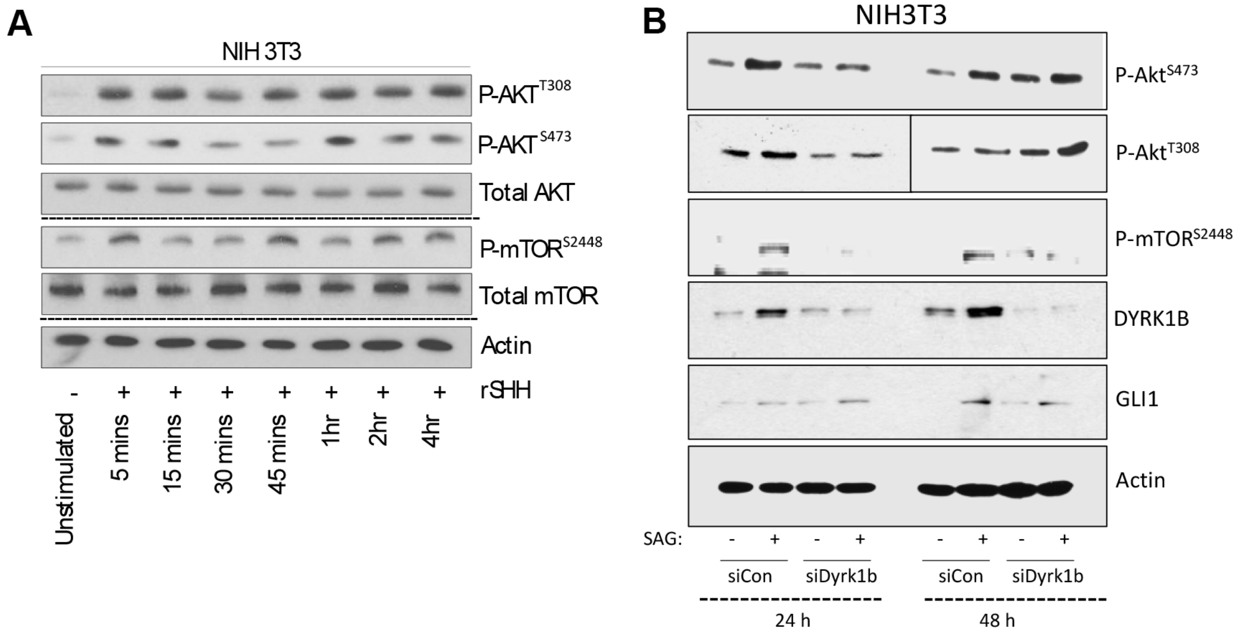

Supplementary Figure S4: Hedgehog-induced AKT/mTOR phosphorylation. (**A**) Detection of phospho-AKT and phospho-mTOR in NIH3T3 cells exposed to various times of recombinant SHH (rSHH (C24II, R&D Systems), 0.4 µg/ml) in 0.5% FBS. (**B**) Detection of phospho-AKT and phospho-mTOR in siCon/siDyrk1b-transfected NIH3T3 cells. Following two transfection rounds on two successive days, cells were treated with SAG (100 nM; 0.5% FBS; third day) for 24 h or 48 h before lysis and Western blotting.

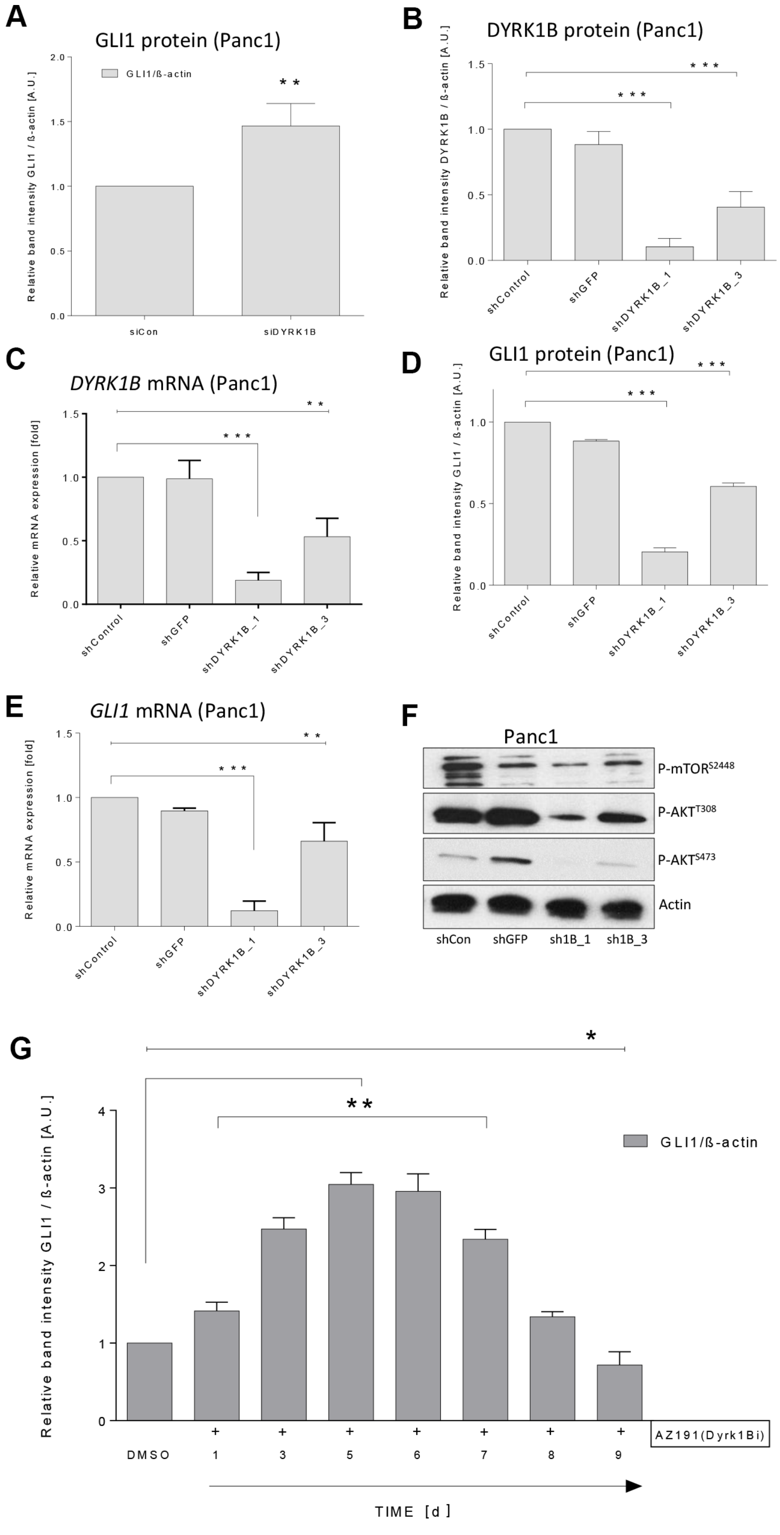

Supplementary Figure S5: Short versus long-term *DYRK1B* knock-down in Panc1 cells. (**A**) Quantification of GLI1 protein levels after acute *DYRK1B* knock-down with siRNA in Panc1 cells. The corresponding WB is depicted in Figure 5D. (**B**) Quantification of DYRK1B protein levels after long-term *DYRK1B* knock-down with shRNA in Panc1 cells. A corresponding WB is depicted in Figure 5E. (**C**) Quantification of *DYRK1B* mRNA levels after long-term *DYRK1B* knock-down with shRNA in Panc1 cells. A corresponding WB is depicted in Figure 5E. (**D**) Quantification of GLI1 protein levels after long-term *DYRK1B* knock-down with shRNA in Panc1 cells. A corresponding WB is depicted in Figure 5E. (**E**) Quantification of *GLI1* mRNA levels after long-term *DYRK1B* knock-down with shRNA in Panc1 cells. A corresponding WB is depicted in Figure 5E. (**F**) Immunoblot depicting the levels of phospho-AKT and phosho-mTOR phosphorylation in Panc1 cells transfected with two different *DYRK1B*-specific shRNAs (*sh1B_1, sh1B_3*) or with two different control shRNA plasmids (*shCon, shGFP*). Cells were harvested 7d after transfection. This panel relates to Figure 5E. (**G**) Quantification of GLI1 protein levels after pharmacological inhibition of DYRK1B using the small molecule AZ191 in Panc1 cells. The corresponding WB of this time-course experiment is depicted in Figure 5F.

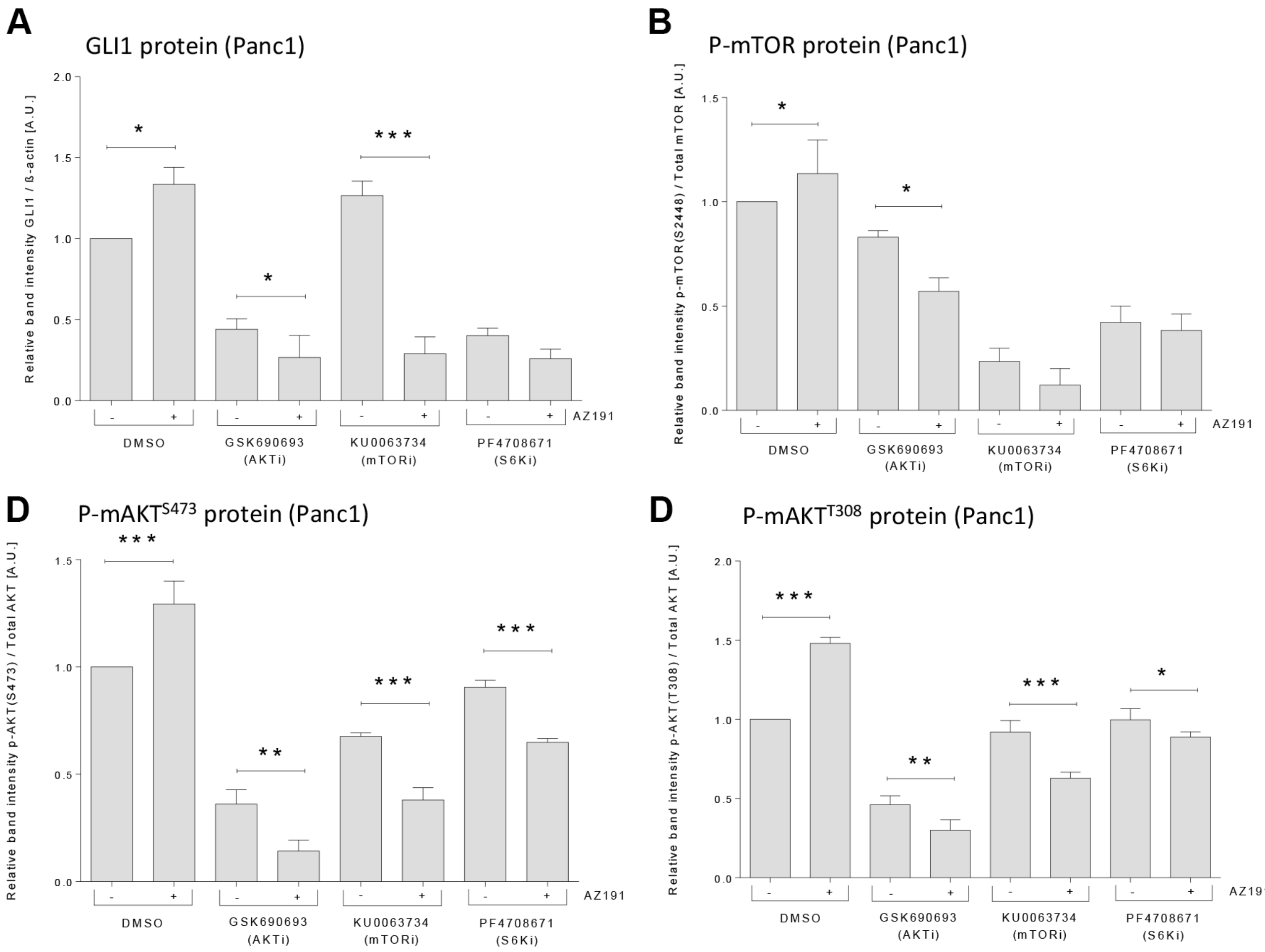

Supplementary Figure S6: DYRK1B inhibition and its effects on GLI1 and AKT/mTOR phosphorylation. Quantification of n = 3 independent blots relating to figure 6A (Panc1 treated with combinations of inhibitors for 24 h). (**A**) GLI1 protein levels. (**B**) Phospho-mTOR levels. (**C**) Phospho-AKT^{S473} levels. (**D**) Phospho-AKT^{T308} levels.

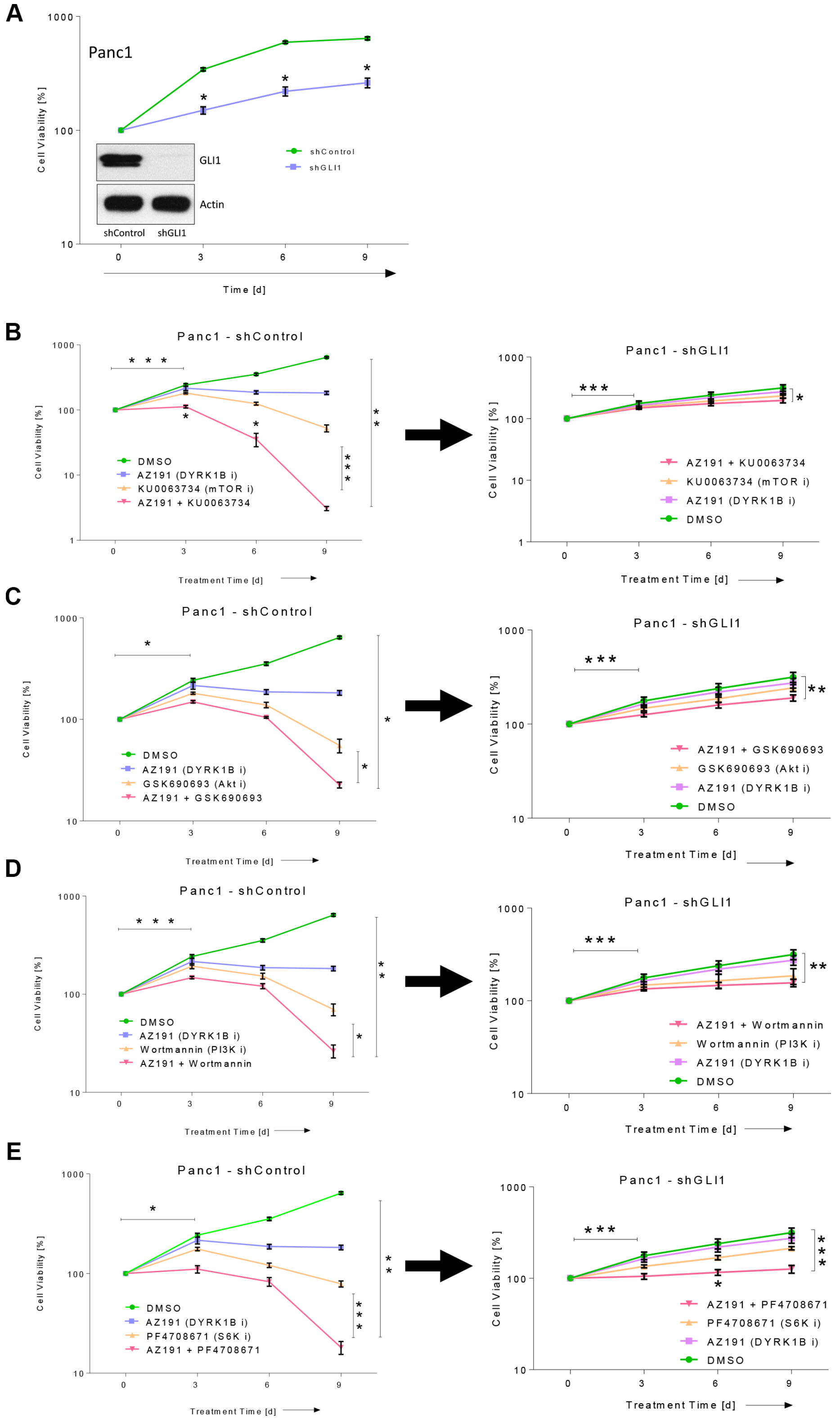

Supplementary Figure S7: Depletion of GLI1 reduces cell growth effects of PI3K/AKT/mTOR inhibitors. (**A**) Growth curve of Panc1 cells (0.5% FBS) transfected with the indicated shRNA constructs. Significances were calculated between corresponding time points of the shCon versus the shGLI1 data points. The inset depicts a Western blot of Panc1 lysates harvested 9 d after transfection with the indicated shRNAs. (**B**) Growth curve of Panc1 cells (0.5% FBS) transfected with the indicated shRNA constructs and treated with AZ191 plus/minus mTOR inhibitor (mTORi). Left panel: shCon-transfected cells. Right panel: shGLI1-transfected cells. (**C**) Growth curve of Panc1 cells (0.5% FBS) transfected with the indicated shRNA constructs and treated with AZ191 plus/minus AKT inhibitor (AKTi). Left panel: shCon-transfected cells. Right panel: shGLI1-transfected cells. (**D**) Growth curve of Panc1 cells (0.5% FBS) transfected with the indicated shRNA constructs and treated with AZ191 plus/minus PI3K inhibitor (PI3Ki). Left panel: shCon-transfected cells. Right panel: shGLI1-transfected cells. (**E**) Growth curve of Panc1 cells (0.5% FBS) transfected with the indicated shRNA constructs and treated with AZ191 plus/minus S6K inhibitor (S6Ki). Left panel: shCon-transfected cells. Right panel: shGLI1-transfected cells.

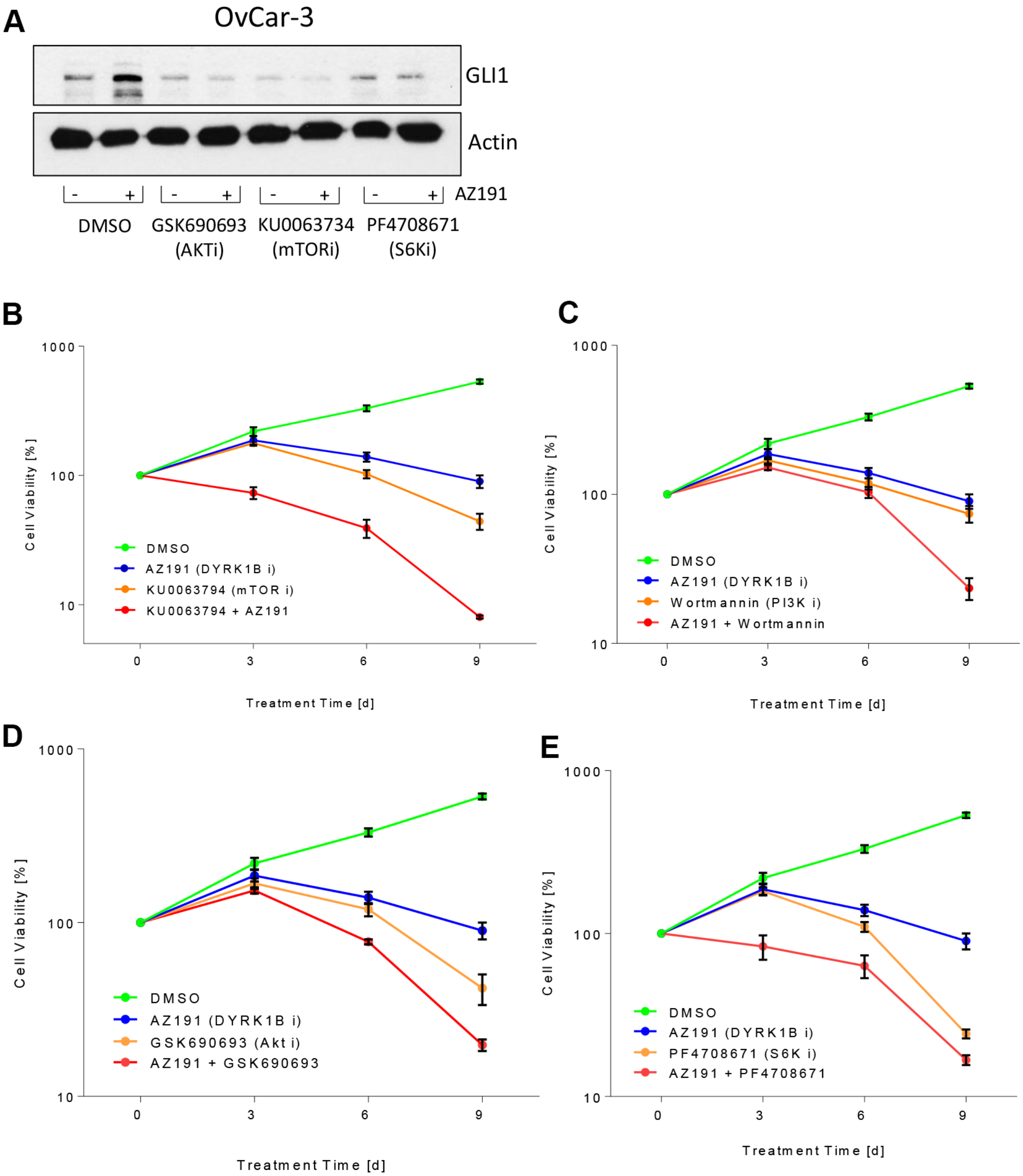

Supplementary Figure S8: Dual targeting of DYRK1B and PI3K/mTOR/AKT in ovarian cancer cells. (**A**) Western blot of OvCar-3 cells treated with the indicated combinations of inhibitors for 24 h in 0.5% FBS. (**B**) OvCar-3 cell growth curve (mean ± SD of *n* = 3). Cells were treated with DMSO, AZ191 (1 µM), KU-0063794 (dual mTORC1/2 inhibitor; 1 µM) as indicated. (**C**) OvCar-3 cell growth curve (mean ± SD of *n* = 3). Cells were treated with DMSO, AZ191 (1 µM), Wortmannin (PI3K inhibitor; 1 µM) as indicated. (**D**) OvCar-3 cell growth curve (mean ± SD of *n* = 3). Cells were treated with DMSO, AZ191 (1 µM), GSK-690693 (pan-AKT inhibitor; 10 µM) as indicated. (**E**) OvCar-3 growth curve (mean ± SD of *n* = 3). Cells were treated with DMSO, AZ191 (1 µM), PF-4708671 (S6K1 inhibitor; 10 µM) as indicated.
